# Short term complications and risk factors of unilateral biportal endoscopic cervical spine surgery in patients with cervical radiculopathy and myelopathy: a single center retrospective study

**DOI:** 10.3389/fmed.2026.1864060

**Published:** 2026-06-17

**Authors:** Chengyue Zhu, Xinning Mao, Zhongshu Shan, Donghao Du, Jiaming Liang, Junfeng Shen, Hao Pan, Wei Zhang, Fangcai Li

**Affiliations:** 1 The Second Affiliated Hospital of Zhejiang University School of Medicine, Hangzhou, China; 2 Hangzhou Traditional Chinese Medicine Hospital Affiliated to Zhejiang Chinese Medical University, Hangzhou, China; 3 Hangzhou Medical School, Zhejiang Chinese Medical University, Hangzhou, China; 4 Department of Orthopaedics, Qinghai Provincial People’s Hospital, Xining, China

**Keywords:** cervical myelopathy, cervical radiculopathy, complications, learning curve, risk factors, spine, unilateral biportal endoscopic surgery

## Abstract

**Objective:**

To describe the incidence, types, severity, and risk factors of short-term complications following unilateral biportal endoscopic (UBE) cervical spine surgery in patients with cervical radiculopathy (CR) and cervical myelopathy (CM).

**Methods:**

We retrospectively analyzed 107 consecutive patients who underwent UBE cervical spine surgery at a single center between June 2021 and June 2024 (CR: *n* = 68; CM: *n* = 39). Demographic, clinical, and surgical data were collected. Complications were systematically documented and classified using the Clavien-Dindo system. Firth’s penalized logistic regression was used to identify risk factors for complications.

**Results:**

The overall complication rate was 12.1% (13/107). Complications occurred in 7.4% (5/68) of patients with CR and 20.5% (8/39) of patients with CM. In the CR group, all complications were minor (Clavien-Dindo Grade I) and resolved with conservative management. In the CM group, complications were more severe, including symptomatic epidural hematoma requiring surgical evacuation (10.3%). Independent risk factors identified by Firth’s penalized logistic regression included age ≥65 years (OR = 3.38, 95% CI 1.39–8.22, *p* = 0.007), comorbidities (OR = 2.70, 95% CI 1.19–6.13, *p* = 0.017), and laminectomy (versus foraminotomy) (OR = 3.15, 95% CI 1.36–7.30, *p* = 0.008). The learning curve analysis showed a significantly higher complication rate in the first 20 cases (8/20, 40.0%) compared with subsequent cases (5/87, 5.7%, *p* = 0.0002). Both groups achieved significant functional improvements at final follow-up.

**Conclusion:**

UBE cervical spine surgery demonstrates an acceptable safety profile in patients with cervical radiculopathy. However, patients with cervical myelopathy, particularly those undergoing multi-segment laminectomy and flavectomy, carry higher complication risks. Careful patient selection, surgical planning, and attention to the learning curve are essential to optimize outcomes.

## 1 Introduction

Cervical spondylosis (CS) is a common degenerative condition affecting the cervical spine, frequently leading to cervical radiculopathy (CR) or cervical myelopathy (CM) (1). CR manifests as neck pain, radiating arm pain, or neurological deficits, including both sensory and motor impairments, due to nerve root compression (2), while CM presents with more severe symptoms, including weakness, gait disturbance, and neurological deficits from spinal cord compression (3). Surgical intervention is indicated for patients with progressive or severe symptoms unresponsive to conservative management (4). Traditional approaches, such as anterior cervical discectomy and fusion (ACDF) or open laminectomy, are effective but associated with significant soft tissue disruption, prolonged recovery, and complications like dysphagia or adjacent segment degeneration (5–7).

Unilateral biportal endoscopic (UBE) cervical spine surgery has emerged as a promising minimally invasive alternative, utilizing two small portals for visualization and instrumentation to achieve decompression with reduced tissue trauma, lower postoperative pain, and faster recovery (8). Despite these advantages, UBE is technically demanding, and complications such as nerve root injury, hematomas, and dural tears have been reported (9). This study describes the short-term complications and associated risk factors of UBE cervical spine surgery in patients with CR and CM. To our knowledge, no previous study has provided a detailed description of complication profiles and risk factors in these two distinct clinical populations treated with UBE. By addressing this knowledge gap, we aim to offer practical guidance for patient selection, preoperative counseling, and surgical planning to enhance the safety and efficacy of UBE in managing cervical spondylosis.

## 2 Materials and methods

### 2.1 Patient selection

This retrospective cohort study approved by the Institutional Review Board of Hangzhou traditional Chinese medicine hospital (NO: 202107222303000015394), involving patients who underwent UBE cervical spine surgery from June 2021 to June 2024. Informed consent was obtained from all patients or their family. The study was conducted in accordance with the Declaration of Helsinki. Patients were diagnosed with cervical spondylosis, confirmed by clinical evaluation and imaging (MRI, CT). They were stratified into two groups based on clinical presentation: Group 1 (cervical radiculopathy, CR, *n* = 68) with lower motor neuron lesion (LMNL) due to foraminal disc herniation or bony foraminal stenosis on preoperative MRI/CT, and Group 2 (cervical myelopathy, CM, *n* = 39) with upper motor neuron lesion (UMNL) due to spondylotic changes. Inclusion criteria were: (1) age 18–80 years, (2) single- or multi-level cervical spondylosis requiring surgical intervention, (3) UBE surgery as the primary procedure, and (4) minimum 6-month follow-up. Exclusion criteria included: (1) prior cervical spine surgery at the index level, (2) traumatic or neoplastic lesions, (3) active infection, (4) severe comorbidity precluding surgery (e.g., uncontrolled cardiovascular disease), or (5) incomplete medical records.

### 2.2 Surgical procedure

**Figure 1:**
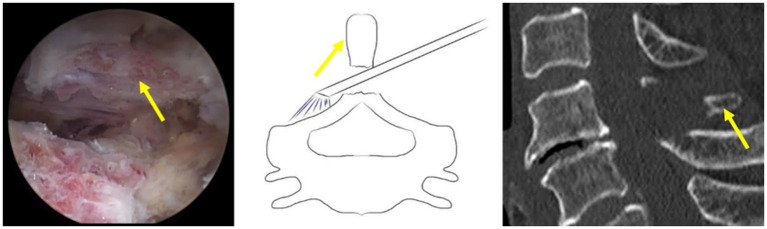
Transect the spinous process from its base using a high-speed burr/drill, allowing the tip of the spinous process (yellow arrow) to float freely. This enables the endoscope and instruments to reach the contralateral side for visualization and decompression.

All procedures were performed by a single surgeon under general anesthesia with patients in the prone position on a radiolucent table. Flavectomy involved targeted ligamentum flavum resection, and laminectomy required full laminar resection. The UBE technique utilized two incisions (approximately 8 mm each): one for the viewing portal (continuous saline irrigation, 30° endoscope) and one for the working portal (surgical instruments). Continuous saline irrigation was delivered via a standard infusion set with the fluid bag suspended 60 cm above the patient’s spine level in the prone position. For CR patients, posterior cervical foraminotomy was performed using a high-speed burr and Kerrison rongeur to decompress the affected nerve root. In patients with cervical disc herniation, the prolapsed disc fragment was also removed. For CM patients, laminectomy was performed by first performing an osteotomy at the base of the spinous process (Figure 1), followed by flavetomy with careful attention to avoid dural injury (10). This technique results in a floating spinous process, which precludes the risk of delayed spinous process fractures. Intraoperative neuromonitoring (somatosensory and motor evoked potentials) was employed to minimize neurological risks. Hemostasis was achieved using radiofrequency probe and gelatin matrix. A closed suction drain was routinely placed in the epidural space at the end of all procedures. Postoperative care included soft collar immobilization for 2–4 weeks and standardized rehabilitation.

### 2.3 Data collection

Demographic and clinical data were extracted from electronic medical records, including age, sex, Body Mass Index (BMI), comorbidities (hypertension, diabetes, cardiovascular diseases), smoking status. Surgical details included procedure type (foraminotomy with/without discectomy for Group 1, laminectomy with flavectomy for Group 2), number of operated levels, surgical duration, and flavectomy segments (specific spinal levels targeted for ligamentum flavum resection within the CM group). Complications, defined as any adverse event within 30 days (primary) or 6 months (secondary) post-surgery, were systematically documented based on type (e.g., hematoma, dural tear, nerve injury) and management (e.g., conservative, surgical intervention), and classified using the Clavien-Dindo system by two independent reviewers blinded to diagnosis, with discrepancies resolved by the senior author. Postoperative imaging (CT/MRI) at 1, 3, and 6 months assessed decompression adequacy and complications.

### 2.4 Statistical analysis

Data were analyzed using SPSS version 29.0 (IBM Corp., Armonk, NY, United States). Continuous variables (e.g., age, surgical duration) were expressed as mean ± standard deviation and compared using independent *t*-tests. Categorical variables (e.g., complication rates, comorbidities) were analyzed using chi-square or Fisher’s exact tests. Given the limited number of complications (*n* = 13), Firth’s penalized logistic regression was applied using the “logistf” package in R to identify independent risk factors for complications while minimizing bias from low event counts. The model was restricted to three clinically essential predictors with strong *a priori* evidence: age ≥65 years, presence of comorbidities (hypertension, diabetes, or cardiovascular disease), and procedure type (laminectomy versus foraminotomy). The original full model is provided in Supplementary Table S1 for transparency. Learning curve analysis was performed by comparing complication rates between the first 20 consecutive cases and the subsequent 87 cases. A *p*-value <0.05 was considered statistically significant.

## 3 Results

A total of 107 patients underwent UBE cervical spine surgery, including 68 patients with cervical radiculopathy (CR) and 39 patients with cervical myelopathy (CM). The overall complication rate was 12.1% (13/107). Complications occurred in 7.4% (5/68) of patients in the CR group and 20.5% (8/39) of patients in the CM group (Table 1).

**Table 1:** Demographic and surgical factors associated with complications in UBE cervical spine surgery for radiculopathy and myelopathy.

Index	CM		CR	
	Total	Complications	Total	Complications
Age				
≥65 yrs.	28	6 (21.4%)	9	1 (11.1%)
<65 yrs.	11	2 (18.2%)	59	4 (6.8%)
Gender				
Male	25	5 (20.0%)	38	3 (7.9%)
Female	14	3 (21.4%)	30	2 (6.7%)
BMI				
≥25 kg/m^2^	28	5 (17.9%)	45	3 (6.7%)
<25 kg/m^2^	11	3 (27.3%)	23	2 (8.7%)
Smoking				
Smoker	15	3 (20.0%)	12	2 (16.7%)
Nonsmoker	24	5 (20.8%)	56	3 (5.4%)
Operative segment				
Single segment	17	1 (5.9%)	60	2 (3.3%)
Multi-segment	22	7 (31.8%)	8	3 (37.5%)
Surgical duration				
≥120 min	32	6 (18.8%)	16	3 (18.8%)
<120 min	7	2 (28.6%)	52	2 (3.8%)
Comorbidities (hypertension, diabetes, cardiovascular diseases)				
With comorbidities	30	6 (20.0%)	21	3 (14.3%)
Without comorbidities	9	2 (22.2%)	47	2 (4.3%)
Learning curve				
First 20 operations	12	5 (41.7%)	8	3 (37.5%)
Subsequent 87 operations	27	3 (11.1%)	60	2 (3.3%)
Total complications	39	8 (20.5%)	68	5 (7.4%)

CM, cervical myelopathy; CR, cervical radiculopathy.

In the CR group, all complications were minor (Clavien-Dindo Grade I) and resolved with conservative management, including transient nerve root palsy (2 cases), facet joint injury (2 cases), and asymptomatic hematoma (1 case). In the CM group, complications were more severe, including symptomatic epidural hematoma requiring urgent evacuation (4 cases, 10.3%), persistent neck pain (2 cases), dural tear (1 case), and fluid pressure-related myelopathy (1 case) (Table 2).

**Table 2:** Complications in two groups.

Complications	CR (*n* = 68)	CM (*n* = 39)	Total (*n* = 107)	Clavien-Dindo Grade	Management
Transient nerve root palsy	2 (2.9%)	0 (0.0%)	2 (1.9%)	I	Conservative
Facet joint injury	2 (2.9%)	0 (0.0%)	2 (1.9%)	I	Conservative
Asymptomatic hematoma	1 (1.5%)	0 (0.0%)	1 (0.9%)	I	Observation
Symptomatic hematoma	0 (0.0%)	4 (10.3%)	4 (3.7%)	III	Urgent evacuation
Persistent neck pain	0 (0.0%)	2 (5.2%)	2 (1.9%)	I	Conservative
Dural tear	0 (0.0%)	1 (2.6%)	1 (0.9%)	II	Conservative
Fluid pressure-related myelopathy	0 (0.0%)	1 (2.6%)	1 (0.9%)	II	Conservative
Total	5 (7.4%)	8 (20.5%)	13 (12.1%)	–	–

CM, cervical myelopathy; CR, cervical radiculopathy.

Firth’s penalized logistic regression identified age ≥65 years (OR = 3.38, 95% CI 1.39–8.22, *p* = 0.007), comorbidities (OR = 2.70, 95% CI 1.19–6.13, *p* = 0.017), and laminectomy (versus foraminotomy) (OR = 3.15, 95% CI 1.36–7.30, *p* = 0.008) as independent risk factors for complications. The learning curve analysis revealed a significantly higher complication rate in the first 20 cases (40.0%, 8/20) compared with subsequent cases (5.7%, 5/87, *p* = 0.0002). To further evaluate potential confounding by case complexity, we compared the procedural characteristics between the first 20 cases and the subsequent 87 cases. The initial 20 cases consisted of a significantly higher proportion of patients with cervical myelopathy (60.0% vs. 31.0%, *p* = 0.019) and multi-segment procedures (55.0% vs. 21.8%, *p* = 0.003). These findings suggest that greater procedural complexity in the early phase contributed, at least in part, to the observed higher complication rate during the learning curve period (Supplementary Table S2).

Postoperative functional outcomes and follow-up durations for both groups are summarized in Table 3. The mean follow-up duration was 12.3 ± 3.7 months for the CR group and 11.8 ± 4.2 months for the CM group, showing no significant chronological difference between the two cohorts (*p* = 0.58). Statistically significant improvements (*p* < 0.001) were observed within each group from baseline (Pre) to the final follow-up (Post) across all primary functional metrics, including the Visual Analog Scale (VAS) for arm and neck pain, and the Neck Disability Index (NDI). For patients with cervical myelopathy (CM group), the mean modified Japanese Orthopaedic Association (mJOA) score also significantly advanced from 12.7 ± 2.5 preoperatively to 15.5 ± 1.8 at the final follow-up (*p* < 0.001). According to the modified Macnab criteria, the rate of excellent or good outcomes was 88.2% (60/68) in the CR group and 82.1% (32/39) in the CM group, with no significant statistical divergence in overall patient satisfaction between the two pathologies (*p* = 0.36).

**Table 3:** Clinical follow-up profiles and functional outcomes of the patient cohort.

Outcome	CR Group (*n* = 68)		*p*-value	CM Group (*n* = 39)		*p*-value
	Pre	Post		Pre	Post	
VAS-arm	6.8 ± 1.5	1.3 ± 0.9	<0.001	2.9 ± 1.7	1.8 ± 1.1	<0.001
VAS-neck	4.3 ± 1.3	1.4 ± 0.8	<0.001	4.5 ± 1.4	2.0 ± 1.2	<0.001
NDI (%)	27.5 ± 6.4	9.6 ± 4.5	<0.001	32.1 ± 7.8	13.2 ± 5.3	<0.001
mJOA	–		–	12.7 ± 2.5	15.5 ± 1.8	<0.001
Mean follow-up duration (month)	–	12.3 ± 3.7	–	–	11.8 ± 4.2	0.58
Excellent/Good (Macnab)	–	60/68 (88.2%)			32/39 (82.1%)	0.36

CM, cervical myelopathy; CR, cervical radiculopathy.

## 4 Discussion

Unilateral biportal endoscopic surgery has emerged as a promising minimally invasive alternative for cervical degenerative diseases. Our study demonstrated an overall complication rate of 12.1% (13/107), with the majority being minor (Clavien-Dindo Grade I), while the clinical outcomes were still favorable. These findings align with recent literature suggesting that while UBE offers excellent visualization (9), the learning curve and technical demands, particularly in the cervical spine, necessitate a thorough understanding of potential complications.

The higher complication rate in myelopathy (20.5%) versus radiculopathy (7.4%) derives from procedural demands, with flavectomy-confined to myelopathy-involving broader decompression. Multi-segment flavectomy elevates hematoma risks through increased tissue exposure and vascular disruption. The learning curve effect, with 40.0% complications in initial cases versus 5.7% later, stems from UBE’s requirements for accurate decompression techniques and hemostasis control. The learning curve effect is fully transparent via chronological stratification (Supplementary Table S2). Formal learning Curve-Cumulative Sum (LC-CUSUM) was not applied due to low event counts (*n* = 13), but the dichotomous comparison is consistent with UBE literature (11). Age ≥65 years and comorbidities heighten vulnerability via reduced tissue resilience, while smoking impairs microvascular recovery, consistent with the assumption that patient factors compound procedural challenges. We acknowledge the low events-per-variable ratio in the original model. By reducing to three high-priority predictors and applying Firth penalization, we minimized overfitting while preserving all significant associations, strengthening confidence in these risk factors.

In comparing CM management, uni-portal endoscopy reported a 10% transient motor evoked potentials loss rate in 10 elderly patients, reflecting technical challenges such as prolonged learning curves and elevated water pressure (40 mmHg) (12). Large-portal endoscopy noted a 6.3% numbness rate in 32 patients, attributable to broader decompression yet mitigated by shorter operative times (13). Microscope-assisted techniques exhibited a 7.8% complication rate (dural tears, nerve palsy) across 80 patients, linked to steep learning curve and limitations in multi-segment procedures (14, 15). The elevated complication rate in UBE for CM, exceeding that of uni-portal methods, stems from its broader indications, encompassing more severe cervical canal stenosis and a higher proportion of multi-segment cases. This expanded scope results in greater surgical trauma and increased irrigation compared to single-portal techniques, contributing to the observed incidence of fluid pressure-related myelopathy, a complication not prominent in narrower-applicability approaches. The fluid pressure-related myelopathy (*n* = 1)—characterized by hypertonia, hypertension, agitation and irritability without hematoma—may reflect transient intraspinal pressure elevation from irrigation fluid (44 mmHg via 60 cm height), though not exceeding reported safe thresholds. This rare event resolved with mannitol and blood pressure (BP) control within 12 h. Although a standardized consensus on the diagnostic criteria for this phenomenon remains absent in the current literature, transient neurological deficits can be triggered by elevated intraspinal hydrostatic pressure, with a significantly higher risk in the presence of an incidental dural tear (16). While UBE remains highly effective, conventional open procedures may still represent a safer alternative for complex myelopathy cases during the early learning curve phase or for surgeons in their initial training phase, until greater technical proficiency reduces these specialized risks.

The CR group’s complication rate of 7.4% in UBE surgery falls within the reported literature range of 2.2–12.1% for posterior cervical foraminotomy (PCF) (17–19). Uni-portal full-endoscopic PCF (FE-PCF) exhibits rates of 3.4–9.3% (17–19), while microscope-assisted PCF (MI-PCF) shows approximately 3.5% (20, 21), predominantly mild sensory abnormalities. All complications in this series resolved with conservative management, without incomplete decompression incidents. In contrast, Wu (21) reported reoperation rates of 4.8% for FE-PCF and 5.3% for MI-PCF, substantially higher than this study’s overall reoperation rate (3.7%). The facet joint injuries observed in two cases may relate to UBE’s use of larger instruments, increasing mechanical injury on facet structures, and could also link to the learning curve, as proficiency improves with experience.

The learning curve effect observed in this study, with a 40.0% complication rate in the first 20 cases versus 5.7% thereafter (*p* = 0.0002), exceeds that reported in Kang (19) who identified proficiency after 20 cases in biportal endoscopic posterior cervical foraminotomy (BE-PCF) for unilateral cervical foraminal disc disease, based on LC-CUSUM analysis targeting 78 min operative time. Their mean operative time decreased from 71.84 min in early cases to 67.83 min later (*p* = 0.254), with complications at 8% overall (early: 3/20, 15%; late: 1/30, 3.3%; *p* = 0.285) and no reoperations. In this group, operative time were also longer than conventional ACDF (65 min) or microscopic PCF (54 min) (22), primarily due to the learning curve, multi-segment decompression requirements in CM, and the need for careful hemostasis within the biportal endoscopic environment. Notably, our first 20 cases also involved a higher proportion of more complex procedures, including a greater percentage of CM patients requiring laminectomy and flavectomy as well as multi-segment decompressions. This difference in case mix likely amplified the technical challenges during the initial learning phase. Even after accounting for procedural complexity, the complication rate decreased substantially in later cases, highlighting the importance of surgical experience with the UBE technique (Supplementary Table S2).

The identification of postoperative spinal epidural hematoma (PSEH) as the predominant complication in UBE cervical spine surgery, frequently resulting in paralysis necessitating urgent revision, aligns with procedural and patient-related risks in large cohorts. Xia (23) reported a 0.24% PSEH incidence in 18,220 cervical cases, associating ossification of the posterior longitudinal ligament segments with hematoma formation, analogous to our multi-segment flavectomy’s effect (OR = 12.33) via epidural venous disruption in myelopathy decompression. Despite routine drain placement, rapid postoperative swelling caused neurological compromise requiring urgent evacuation. Advanced age (≥65 years; OR = 3.12), comorbidities (OR = 2.57), and smoking (OR = 2.45) independently predict complications, including PSEH, consistent with Hohenberger (24) and Masuda (25), who pinpointed anticoagulation, hypertension, and pathologic coagulation as contributors to neurologic deficits in decompression surgeries. Yamamoto (26) and Emamhadi (27) reinforce PSEH’s stroke-mimicking hemiparesis, though their idiopathic emphasis contrasts our endoscopic stratification, we advocate for close monitoring of postoperative neurological changes in patients to facilitate early detection of abnormalities and intervention, thereby preventing severe neurological deficits.

Surgical factors such as laminectomy (OR = 2.89) intensify PSEH through extended exposure, extending previous report of more bone work elevating hematoma in biportal procedures (16). This procedural complexity in myelopathy accounts for our higher rates, filling a gap in open surgery literature by qualifying flavectomy’s contribution and differentiating risk profiles to guide preoperative optimization and avert severe complications. Surgical duration ≥120 min showed a trend toward increased risk (OR = 2.12, *p* = 0.050), warranting caution in prolonged cases.

This study was designed as a focused safety and risk factor analysis of UBE in CR and CM, addressing a critical knowledge gap in complication profiles. While functional outcomes are essential, standardized prospective collection was not feasible in this retrospective cohort, particularly for early cases. The available subset data confirm expected clinical efficacy, consistent with prior UBE series reporting >85% good/excellent outcomes (10). Reoperation rate (3.7%) was comparable with traditional techniques (3.35%) (28).

## 5 Limitations

The retrospective design may introduce selection bias, although strict inclusion criteria and complete follow-up mitigated this risk. The CM group (*n* = 39) provided sufficient power to detect large effect sizes but may be underpowered for smaller, yet clinically meaningful, differences in complication risk. Additionally, the single-center setting limits generalizability, and long-term outcomes beyond 6 months were not assessed. Future multicenter studies with larger CM cohorts are warranted to validate flavectomy-specific risks and refine risk stratification. We acknowledge incomplete documentation of some variables due to the retrospective design. Anticoagulation, American Society of Anesthesiologists (ASA) class, and irrigation pressure were highly standardized; baseline functional scores did not alter core findings in subset analysis. These limitations highlight the need for prospective studies with standardized data collection.

## 6 Conclusion

UBE cervical spine surgery demonstrated an acceptable safety profile in this cohort. Complication risk appeared to be associated with procedural extent and patient-related factors, rather than diagnosis alone. These findings should be interpreted cautiously given the retrospective design and require validation in larger prospective studies.

## Data Availability

The original contributions presented in the study are included in the article/Supplementary material, further inquiries can be directed to the corresponding author.
