# Supplementary material for: Short term complications and risk factors of unilateral biportal endoscopic cervical spine surgery in patients with cervical radiculopathy and myelopathy: a single center retrospective study
Source: Front Med (Lausanne). 2026 Jun 17;13:1864060. doi: 10.3389/fmed.2026.1864060 (PMC13318707; doi:10.3389/fmed.2026.1864060)
Supplement: Supplementary file 1 [file Table_1.DOCX]

Supplementary Table S1. Original Full Multivariable Logistic Regression Model (for reference only; caution advised due to low EPV)

| **Predictor** | OR (95% CI) | p-value |
| --- | --- | --- |
| **Age ≥65 years** | 3.12 (1.18–8.25) | 0.02 |
| **Comorbidities** | 2.57 (1.09–6.04) | 0.03 |
| **Smoking** | 2.45 (1.04–5.77) | 0.04 |
| **Surgical duration >120 min** | 2.12 (1.00–4.50) | 0.05 |
| **Laminectomy (vs. foraminotomy)** | 2.89 (1.12–7.46) | 0.03 |
