# Supplementary material for: Short term complications and risk factors of unilateral biportal endoscopic cervical spine surgery in patients with cervical radiculopathy and myelopathy: a single center retrospective study
Source: Front Med (Lausanne). 2026 Jun 17;13:1864060. doi: 10.3389/fmed.2026.1864060 (PMC13318707; doi:10.3389/fmed.2026.1864060)
Supplement: Supplementary file 2 [file Table_2.DOCX]

Supplementary Table S2. Comparison of Procedural Complexity Between Early and Late Cases in the Learning Curve

| Variable | First 20 cases | Subsequent 87 cases | p-value |
| --- | --- | --- | --- |
| CM patients, n (%) | 12 (60.0%) | 27 (31.0%) | 0.019 |
| Multi-segment procedure, n (%) | 11 (55.0%) | 19 (21.8%) | 0.003 |
| Laminectomy, n (%) | 12 (60.0%) | 27 (31.0%) | 0.019 |
| Mean surgical duration (min) | 138.5 ± 42.6 | 96.7 ± 35.4 | <0.001 |

|
